# Study on Self-Sharpening Mechanism and Polishing Performance of Triethylamine Alcohol on Gel Polishing Discs

**DOI:** 10.3390/mi16070816

**Published:** 2025-07-16

**Authors:** Yang Lei, Lanxing Xu, Kaiping Feng

**Affiliations:** 1 Special Equipment Institute, Hangzhou Vocational & Technical College, No. 68, Xueyuan Street, Hangzhou 310018, China; leiyangcn@126.com; 2 College of Mechanical Engineering, Quzhou University, No. 78, North Jiuhua Road, Quzhou 324000, China; 221122020197@zjut.edu.cn

**Keywords:** glazing phenomenon, self-sharpening, gel polishing disc, material removal rate, surface roughness

## Abstract

To address the issue of surface glazing that occurs during prolonged polishing with gel tools, this study employs a triethanolamine (TEA)-based polishing fluid system to enhance the self-sharpening capability of the gel polishing disc. The inhibitory mechanism of TEA concentration on disc glazing is systematically analyzed, along with its impact on the gel disc’s frictional wear behaviour. Furthermore, the synergistic effects of process parameters on both surface quality and material removal rate (*MRR*) of SiC are examined. The results demonstrate that TEA concentration is a critical factor in regulating polishing performance. At an optimal concentration of 4 wt%, an ideal balance between chemical chelation and mechanical wear is achieved, effectively preventing glazing while avoiding excessive tool wear, thereby ensuring sustained self-sharpening capability and process stability. Through orthogonal experiment optimization, the best parameter combination for SiC polishing is determined: 4 wt% TEA concentration, 98 N polishing pressure, and 90 rpm rotational speed. This configuration delivers both superior surface quality and desirable *MRR*. Experimental data confirm that TEA significantly enhances the self-sharpening performance of gel discs through its unique complex reaction. During the rough polishing stage, the *MRR* increases by 34.9% to 0.85 μm/h, while the surface roughness *S_a_* is reduced by 51.3% to 6.29 nm. After subsequent CMP fine polishing, an ultra-smooth surface with a final roughness of 2.33 nm is achieved.

## 1. Introduction

Owing to their exceptional hardness and wear resistance, diamond abrasive tools are widely employed in processing difficult-to-machine materials such as silicon carbide and monocrystalline silicon [1,2]. Fixed abrasive tools, particularly diamond-based ones, have become a mainstream precision machining solution for hard and brittle materials due to their superior performance [3,4]. Most commercial diamond copper discs are fabricated by hot pressing. While hot-pressed abrasive tools exhibit high machining efficiency, they often suffer from issues like abrasive agglomeration, poor thermal conductivity, and inferior workpiece surface quality [5].

To balance machining efficiency with surface finish, researchers have developed semi-fixed abrasive tools based on the sol–gel method. This technique involves wet-mixing abrasive powders, gelling them through physical or chemical processes, and sintering to form semi-fixed abrasive tools (gel tools) [6]. The sol–gel approach enables more uniform abrasive distribution and adjustable grain protrusion height. Although this method slightly compromises tool strength, it significantly improves surface quality. Luo et al. [7] fabricated small gel polishing spheres that produced smooth surfaces on seal stones while allowing abrasive recovery and reuse. Kong et al. [8] demonstrated that the sol–gel method achieves superior homogeneity in ultrafine powder mixtures. Lin et al. [9] developed sol–gel (SG) polishing pads for monocrystalline diamond (SCD), observing reduced subsurface damage after polishing. Xue et al. [10] created a novel SG tool with carbon fibre reinforcement that reduced marble surface roughness by 98.6% and enhanced glossiness by 326.7%. Chen et al. [11] designed sol–gel spheres for drag-finishing irregular Shoushan stone, reducing roughness from 1132 nm to 287 nm and increasing glossiness from 6 GU to 34.8 GU. Feng et al. [12,13] employed a polyvinyl alcohol (PVA)–phenolic resin (PF) binary system to fabricate gel tools that achieved a 2.55 nm surface roughness on SiC workpieces.

During machining, debris accumulation and transient high temperatures cause pore clogging and surface glazing, gradually diminishing the tool’s material removal capability [14]. To enhance the self-sharpening of tools, researchers have proposed various solutions. Li et al. [15] used thermochemical etching to increase pore volume, boosting grinding efficiency by 29.6%. Chen et al. [16,17] introduced loose alumina abrasives to augment diamond tool self-sharpening. Nayak et al. [18] developed ice-bonded abrasive polishing (IBAP), enabling self-lubrication and self-dressing at low temperatures, with predictive models for tool wear and interface temperature. Zhu et al. [19] observed that polycrystalline cubic boron nitride (PCBN) wheels achieve self-sharpening through grain fracture and renewal. Chen et al. [20] designed sintered spherical diamond (PD) particles that fragment during magnetorheological polishing, exposing fresh micro-cutting edges and enhancing the self-sharpening property of the tools.

In order to improve the self-sharpening ability of the gel disc, this study develops a self-sharpening diamond gel polishing disc using PVA and PF as matrix materials, with electrolytic copper powder as the functional filler. The polishing system is enhanced by introducing triethanolamine (TEA) as a complexing modifier in the polishing fluid. The hydroxyl and amino groups in TEA molecules selectively coordinate with copper ions released from the disc surface, effectively removing glazing layer and re-exposing embedded diamond abrasive grains. This mechanism significantly suppresses glazing phenomena during polishing while improving the tool’s self-sharpening capability. This paper studies the influence of TEA concentration on the complexation reaction and deeply explores the regulation mechanism of TEA addition amount on the self-sharpening property of gel discs, tribological characteristics, and the surface quality of workpieces.

## 2. Self-Sharpening Mechanism

**Figure 1:**
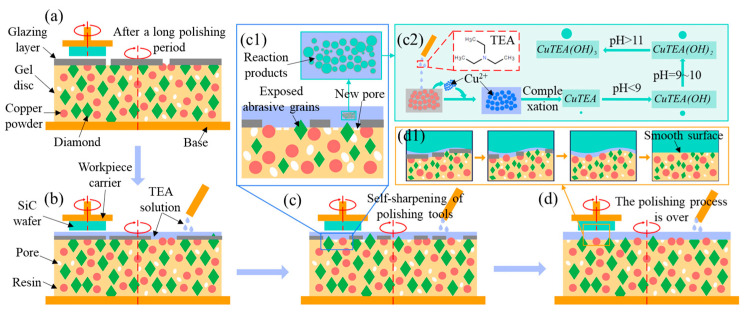
Self-sharpening mechanism of gel polishing discs: (**a**) The disc has been polished for a long time. (**b**) Add TEA polishing solution onto the disc. (**c**) The self-sharpening process of the disc. (c1: The impact of the complex reaction. c2: The products of the complex reaction.) (**d**) The polishing process is over. (d1: The smooth SiC surface is obtained).

Figure 1 illustrates the self-sharpening mechanism of gel polishing discs. During extended polishing operations, the gel polishing tool generates substantial debris primarily consisting of copper particles and dislodged abrasive grains. This debris accumulation leads to pore blockage on the disc surface, causing localized overheating and subsequent glazing phenomena (Figure 1a). The process ultimately forms a dense glazed layer on the abrasive grains, which significantly impairs the gel disc’s material removal capacity and adversely affects machining efficiency. To counteract this issue, the present study employs triethanolamine (TEA) solution as the polishing fluid (Figure 1b). Under alkaline polishing conditions, copper particles on disc surface undergo oxidation, enabling the nitrogen atoms in TEA molecules to coordinate with copper ions (Cu^2+^) through lone electron pair donation. This complexation reaction produces soluble blue or blue-to-greenish coordination compounds (Figure 1c), effectively dissolving the adhered copper debris and consequently reducing the coverage area of the glazed layer. As a result, previously covered abrasive grains become re-exposed to the disc surface. The TEA-Cu^2+^ complexation reaction simultaneously generates numerous micro-pores within the abrasive tool matrix, strategically weakening the matrix’s retention force on the abrasive grains. This controlled weakening facilitates the detachment of worn grains while promoting continuous exposure of new, sharp grains, thereby significantly enhancing the tool’s self-sharpening capability. Through the synergistic combination of TEA’s chemical action and mechanical polishing, the study successfully mitigates the machining efficiency degradation caused by glazing phenomena. This method enables the gel polishing discs to have good self-sharpening performance and the surface quality of the workpiece after polishing is better (Figure 1d).

## 3. Experimental Set-Up

### 3.1. Manufacturing Process of Polishing Disc

**Figure 2:**
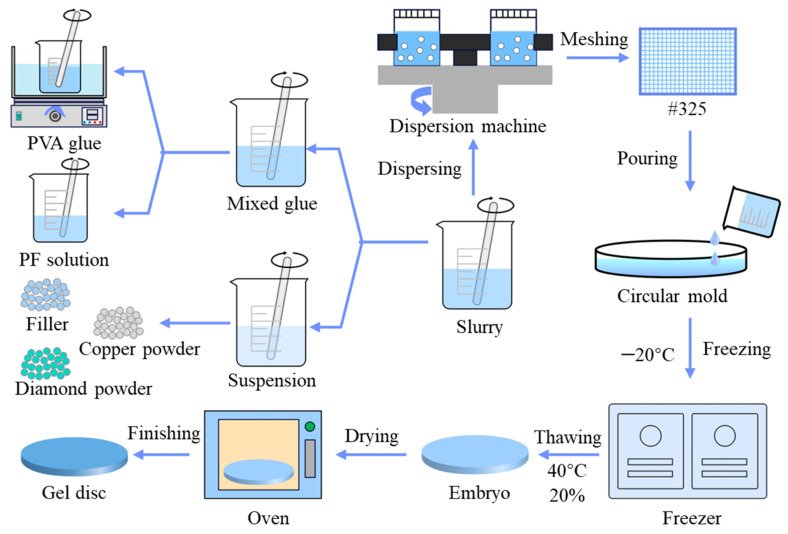
Fabrication process of polishing disc.

The fabrication process and compositional formulation of the gel polishing discs are illustrated in Figure 2 and Table 1, respectively. Alumina powder can be utilized to modulate the friction coefficient of the gel disc, thereby enhancing its frictional properties and mitigating the glazing phenomenon. The manufacturing procedure involves the following sequential steps: First, a predetermined quantity of polyvinyl alcohol (PVA) powder is added to deionized water and continuously stirred at 90 °C in a water bath until complete dissolution, yielding a homogeneous and transparent PVA colloidal solution. This solution is then mixed with a pre-determined ratio of water-soluble resin and mechanically stirred for 30 min to obtain a uniform hybrid colloid. Concurrently, diamond particles, electrolytic copper particles, and other functional additives are dispersed in deionized water according to the designed formulation and subjected to ultrasonic treatment to form a stable suspension system. The mixed colloid is gradually incorporated into the suspension system using a mechanical stirrer operating at 600 rpm for 1 h to produce a homogeneous composite slurry. To ensure slurry quality, the mixture undergoes vacuum stirring (TMV-1500t, Shenzhen, China) for 30 min followed by filtration through a #325-mesh (45 μm pore size) stainless steel sieve to remove particle agglomerates. The refined slurry is then poured into pre-treated circular moulds, which are immediately sealed and transferred to a −20 °C freezer for 6 h of directional freezing, followed by 12 h of natural thawing at 25 °C. The thawed gel bodies are placed in a programmable constant temperature and humidity chamber (maintained at 40 ± 1 °C with 20 ± 5% relative humidity) for 12 h of gradient drying. Finally, gel polishing discs are obtained by holding at 160 °C for 2 h in a programmed temperature-controlled sintering furnace. The sintered discs are then precision-finished using surface grinding machines to produce gel polishing discs with optimal surface morphology and mechanical properties.

### 3.2. Material Characterization

The experimental setup employs the following instrumentation and analytical techniques: A thermostatic water bath is precisely controlled using a heating magnetic stirrer (DF-1, Suzhou Weier Laboratory Products Co., Ltd., Suzhou, China). Surface morphology characterization of polishing disc samples is conducted via field emission scanning electron microscopy (SU8010, Hitachi High-Technologies Corporation, Hitachi, Japan). Bis(cyclohexanone)oxaldihydrazone (BCO) will react with copper ions to form a complex. BCO is used as the chromogenic agent, and the absorbance of the complex is measured by the ultraviolet–visible spectrophotometer (UV1901PC, Shanghai, China). The friction coefficient of the disc samples is tested by using the high-temperature friction and wear testing machine of LanZhou Zhongke Kai Technology Co., Ltd. (HT 1000, Lanzhou, China). The friction pair used in the testing machine is a 5 mm diameter silicon carbide ball, with a normal load of 5N and a centre diameter of the wear mark of 6 mm. Friction ring widths on gel discs are quantified using a Keyence digital microscope (VHX-5000, Shanghai, China). The surface topography of polished SiC workpieces is quantitatively characterized using a non-contact optical profilometer (KLA Tencor MicroXAM 1200, Taiwan, China), which provided surface roughness (Sa) measurements with nanometre-level resolution. Mass variation during polishing is precisely determined using an FA124C electronic analytical balance (Li-Chen, Shenzhen, China) with a measurement accuracy of ±0.1 mg.

### 3.3. Polishing Experiment

**Figure 3:**
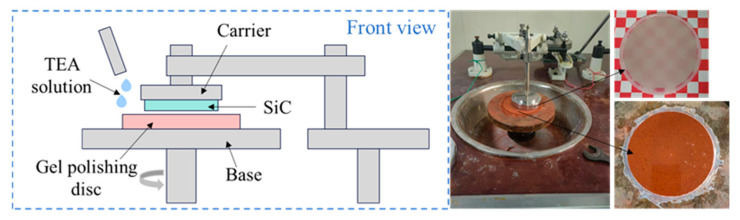
Schematic diagram of polishing device.

This study utilizes 4H-HPSI SiC wafer substrates provided by Shanghai Jingdian New Materials Technology Co., Ltd. (Shanghai, China). Prior to experimentation, all samples underwent precision grinding pretreatment, with initial surface roughness (*S_a_*) controlled at approximately 100 nm. The polishing orientation face is silicon face. The polishing experiments are conducted using a four-axis polishing machine manufactured by XiBin Photo-Electronics Co (JP25.4B, Wuxi, China). Schematic diagram is shown in Figure 3. During the polishing process, a TEA-based polishing solution containing 0.1 wt% hydrogen peroxide (pH adjusted to 10) is continuously applied. In this chemical environment, the copper on the polishing disc surface undergoes oxidation, releasing copper ions that form complexes with TEA. This complex reaction effectively inhibits glazing phenomena while promoting both the exposure of abrasive grains and the removal of passivated particles, thereby significantly enhancing the self-sharpening capability of the polishing disc.

The experimental design comprised two phases: First, process parameters are optimized through orthogonal experiments with factor levels as detailed in Table 2. Subsequently, a systematic evaluation of the gel disc’s self-sharpening improvement is conducted using conventional water-based polishing fluid as a control, with specific experimental parameters listed in Table 3. The experimental parameters are determined based on preliminary research findings. The *MRR* is calculated using Equation (1), where Δ*m* represents the mass difference before and after polishing (g), *ρ* denotes the theoretical density of silicon carbide (3.2 g/cm^3^), *t* is the polishing time (min), and *s* represents the polishing area (cm^2^).

(1) $$MRR=\frac{∆m}{\rho ts}$$

## 4. Results and Discussion

### 4.1. Effect of TEA Content on Glazing Phenomenon

**Figure 4:**
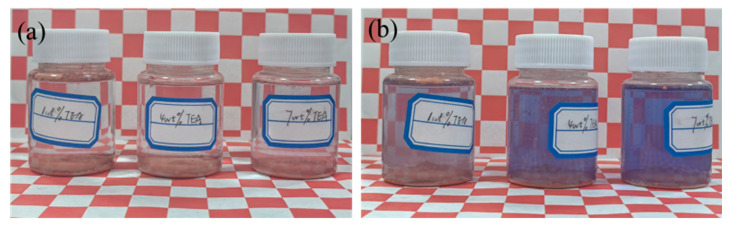
The colour changes in solutions with different TEA polishing solution before and after reacting with copper powder: (**a**) 0 min (**b**) 60 min.

**Figure 5:**
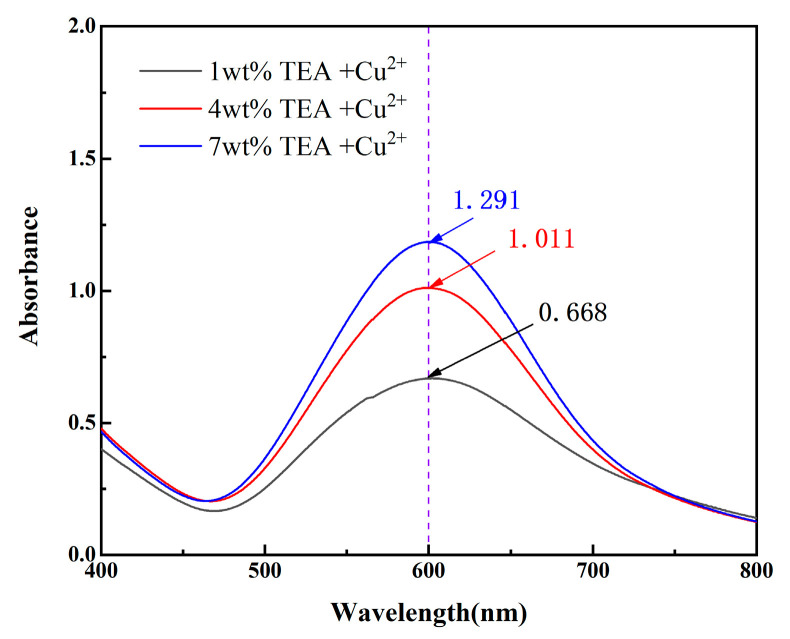
Changes in solution absorbance under different TEA polishing solutions.

Figure 4a presents the initial state of copper powder in different TEA solutions. Figure 4b shows the TEA solutions after 60 min. These results suggest that when the TEA concentration is sufficient, copper ions from the copper powder can undergo complexation reactions with TEA to form blue-coloured complexes [21]. To investigate the influence of TEA concentration on this reaction, BCO is employed as a chromogenic agent, which forms a coloured complex with copper ions, allowing the copper ion concentration to be quantified by measuring the absorbance of the reaction solution at 600 nm [22]. Ultraviolet–visible spectrophotometry results (Figure 5) reveal that the 1 wt% TEA + Cu^2+^ system exhibited the lowest absorbance (0.668). The absorbance of the 4 wt% and 7 wt% TEA + Cu^2+^ systems are increased by 51.2% and 93.3%, respectively, compared with the 1 wt% TEA + Cu^2+^ system, confirming that a higher TEA concentration significantly enhanced the complexation and generated many complexation compounds.

**Figure 6:**
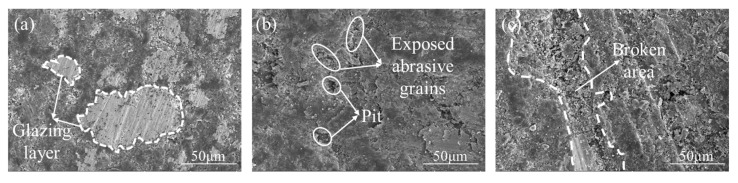
Surface morphology of gel disc after polishing under different TEA concentration polishing solution: (**a**) 1 wt%, (**b**) 4 wt%, (**c**) 7 wt%.

SEM analysis of the gel disc surface after 60 min of polishing (Figure 6) demonstrates distinct morphological changes depending on TEA concentration: At 1 wt% TEA, severe glazing occurs, where a dense glazed layer covers the abrasive grains, reducing mechanical removal efficiency (Figure 6a). When the TEA concentration increases to 4 wt%, complexation reduces the glazed area, exposing more abrasive grains and increasing surface porosity, which weakens grain retention and facilitates a self-sharpening process—where passivated grains detach and fresh abrasives emerge (Figure 6b). However, at 7 wt% TEA, excessive complexation leads to large-scale surface collapse, with a sharp decline in grain retention forming pronounced pits (Figure 6c). Such drastic topographical changes can severely compromise polishing quality.

In summary, TEA concentration exhibits an optimal range for effective polishing: excessively low concentrations (e.g., 1 wt%) induce glazing, while excessively high concentrations (e.g., 7 wt%) cause excessive tool wear. This study identifies 4 wt% TEA as the suitable concentration, striking a balance between complexation and mechanical abrasion to sustain self-sharpening while ensuring consistent surface quality.

### 4.2. Friction and Wear Test

This study systematically examines the influence of TEA concentration (1 wt%, 4 wt%, and 7 wt%) on the friction and wear characteristics of gel copper discs through controlled experiments. By employing real-time tribological monitoring and comprehensive surface morphology analysis, we establish a clear relationship between TEA concentration and the disc’s tribological performance.

**Figure 7:**
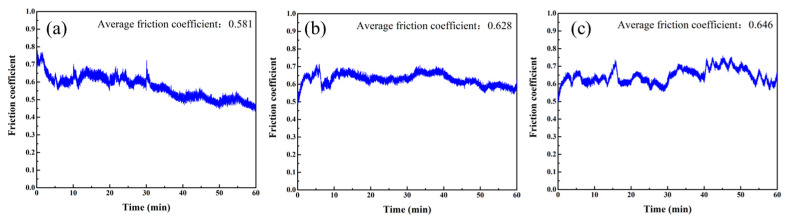
Friction coefficient of gel disc polished under different TEA concentration solution: (**a**) 1 wt%, (**b**) 4 wt%, (**c**) 7 wt%.

As illustrated in Figure 7, the average friction coefficient exhibits an increase with TEA concentration, ranging from 0.581 (1 wt%) to 0.646 (7 wt%). At the lowest concentration (1 wt%), insufficient complexation results in gradual accumulation of debris, leading to pore blockage and the formation of a dense glazed layer. SEM characterization reveals this glazed layer possesses remarkably smooth and compact features, which significantly reduce surface roughness of gel disc (Figure 6a). The morphological change increases the contact area while decreasing shear strength, ultimately manifesting as a low and stable friction coefficient (Figure 7a). However, this surface condition substantially compromises the gel disc’s mechanical removal capability, adversely affecting material removal efficiency and stability. The optimal performance emerges at 4 wt% TEA concentration, where an ideal chemo-mechanical balance is achieved. Moderate complexation effectively prevents glazing while maintaining appropriate abrasive protrusion, resulting in superior friction coefficient stability with minimal reduction (Figure 7b). In contrast, at the highest concentration (7 wt%), excessive complexation disrupts this delicate balance. This intensified chemical reactions severely weaken the holding ability of gel discs, causing massive detachment of copper powder and diamond particles that generates prominent pits and defects on the gel disc surface. This significant surface damage leads to uneven contact stress distribution during friction, inducing substantial coefficient fluctuations (Figure 7c). Furthermore, the enhanced three-body wear effect paradoxically increases the average friction coefficient despite the surface damage.

**Figure 8:**
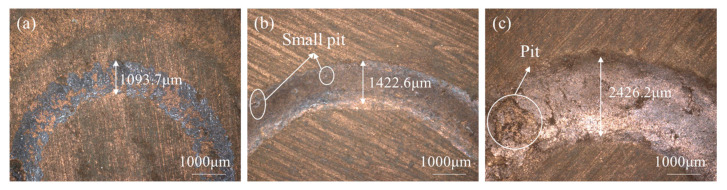
Wear trace of gel disc polished under different TEA concentration solution: (**a**) 1 wt% (**b**) 4 wt%, (**c**) 7 wt%.

Figure 8 presents characteristic wear track morphologies after 60 min of tribological testing. Quantitative analysis using Keyence ultra-depth microscopy (VHX-5000, Shanghai, China) reveals track widths of 1093.7 μm (1 wt%), 1422.6 μm (4 wt%), and 2426.2 μm (7 wt%). Calculated wear volumes using Equations (2) and (3) [12] demonstrate values of 5.52 mm^3^, 9.88 mm^3^, and 27.63 mm^3^, respectively, showing an approximately exponential growth trend with increasing TEA concentration. In-depth analysis of the wear mechanism reveals that the change in TEA concentration mainly affects the wear behaviour through the following pathways. Firstly, under the low TEA concentration condition of 1 wt%, due to the weak complexation reaction between TEA and copper ions, the surface of the polishing disc mainly undergoes uniform wear dominated by mechanical wear. The edge of the friction ring is relatively flat, and the wear depth distribution is uniform (Figure 8a). When the TEA concentration increased to 4 wt%, the enhanced complexation reaction promoted the selective removal of the surface copper matrix, resulting in a transformation of the wear mechanism into a chemical-mechanical synergy. The width of the friction ring increased by approximately 30%, and obvious material transfer characteristics appeared on the surface (Figure 8b). Under the condition of high TEA concentration of 7 wt%, the intense chemical corrosion effect causes a qualitative change in the wear mechanism. The width of the friction ring increases by up to 122% compared to the 1 wt% sample (Figure 8c). The wear surface presents a typical corrosion and wear morphology, and in some local areas, shedding pits with depths exceeding 50 μm can be seen.

In conclusion, an appropriate TEA concentration (4 wt%) can achieve an optimized match between chemical reactions and mechanical wear, avoiding the formation of glazing layers while maintaining good surface integrity. Both excessively high and low concentrations can disrupt this balance, leading to the deterioration of tribological properties.

(2) $$h=r-{\left[r^{2}-{\left(\frac{a}{2}\right)}^{2}\right]}^{\frac{1}{2}}$$

(3) $$V=4\pi b\int_{-r}^{h}\sqrt{r^{2}-x^{2}}dx=2\pi br^{2}\left(\frac{h}{r}\sqrt{1-\frac{h^{2}}{r^{2}}+arcsin\frac{h}{r}+\frac{\pi }{2}}\right)$$

### 4.3. Orthogonal Experiment

**Figure 9:**
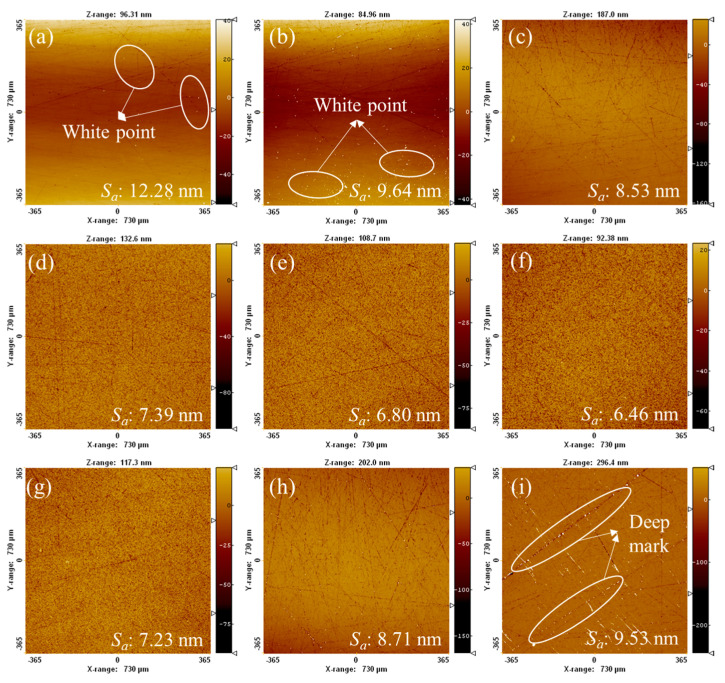
Surface morphology of SiC after polishing with different experimental groups: (**a**–**i**) corresponds to groups 1–9 in Table 4.

This study employs an L9 (3^4^) orthogonal experimental design to systematically evaluate the influence of key process parameters on silicon carbide (SiC) polishing quality. The investigation focuses on three critical factors—triethanolamine (TEA) concentration (A), polishing pressure (B), and platen rotational speed (C)—each examined at three distinct levels as detailed in Table 3. White light interferometry analysis of polished SiC surfaces (Figure 9) demonstrates achievement of surface roughness (*S_a_*) values ranging from 6.46 to 12.28 nm, while maintaining *MRR* between 0.65 and 1.01 μm/h. Utilizing the Taguchi methodology, we implement signal-to-noise ratio (SNR) as the optimization metric, applying “smaller-the-better” characteristics for surface roughness and “larger-the-better” characteristics for *MRR* Equations (4) and (5) [23]. In the formula, *i* is the number of experimental groups, *r* is the number of measurement points (*r* = 5), *μ* is the number of measurements (*μ* = 5), *S* is the *S_a_*, and *M* is the *MRR*.

**Table 4:** Orthogonal experiment results.

Experimental Groups	TEA Content (wt%)	Pressure (N)	Speed (rpm)	Average *S_a_* (nm)	*MRR* μm/h	SNR/dB	
						*S_a_*	*MRR*
1	1	0	30	12.28	0.65	−21.79	6.70
2	1	49	60	9.64	0.84	−19.68	5.81
3	1	98	90	8.54	0.87	−18.63	8.03
4	4	0	60	7.39	0.91	−17.37	8.33
5	4	49	90	6.8	0.96	−16.65	8.72
6	4	98	30	6.46	1.01	−16.21	9.19
7	7	0	90	7.23	0.86	−17.20	9.63
8	7	49	30	8.71	0.81	−18.81	8.26
9	7	98	60	9.53	0.77	−19.60	7.74

**Figure 10:**
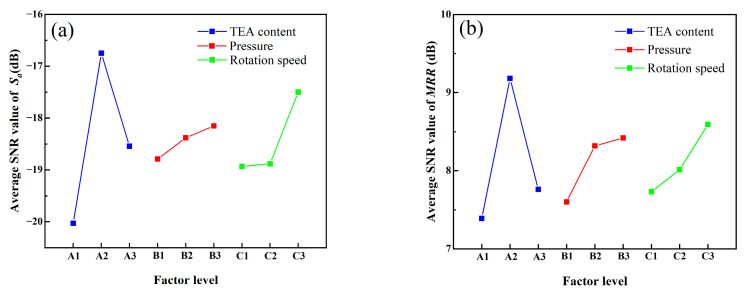
Influence of experimental parameters on polishing effect: (**a**) average SNR value of *S_a_*; (**b**) average SNR value of *MRR*.

Figure 10 shows the average SNR response curves of *S_a_* and *MRR* of SiC samples at different process parameter levels. It can be observed from the figure that the influence of TEA concentration on polishing quality shows significant nonlinear characteristics. When the concentration of TEA is 4 wt%, the average SNR of *S_a_* and *MRR* are the highest. This is because an appropriate amount of TEA can effectively promote the chemical-mechanical synergy on the surface of SiC. At low TEA concentration, the complexation effect is weak, the surface of the gel disc is prone to overheating and glaze, the cutting ability of the abrasive grains decreases during the polishing process, the mechanical removal efficiency drops, and the average SNR of *MRR* decreases by approximately 19.5%. The increase in the glazed area causes some micro-peaks on the SiC surface not to be effectively removed, forming white spot defects on the surface (Figure 9a,b), resulting in a decrease in the average SNR of *S_a_*. When the concentration of TEA is too high, TEA undergoes excessive complexation with the metal ions in the gel disc, reducing the tool’s ability to hold particles and causing a large number of abrasive grains and other particles to fall off. Free abrasive particles generate random micro-cutting during the polishing process, resulting in the deterioration of surface roughness and the decrease in the average SNR of *S_a_* (Figure 9h,i). Meanwhile, due to the reduction in the effective abrasive particle number of the gel disc, the average SNR of *MRR* decreased by 15.5%.

The experimental results in Figure 10 demonstrate that both *S_a_* and *MRR* SNR exhibit a significant increasing trend with rising polishing pressure. When the pressure increases from 0 N to 49 N and 98 N, the SNR of *MRR* improves by 9.5% and 10.8%, respectively. This is because the increase in polishing pressure will expand the actual contact area between the gel disc and the SiC, thereby significantly increasing the number of effective diamond abrasive grains involved in the cutting. Higher pressure increases the penetration depth of a single abrasive grain, thereby enhancing the cutting effect and significantly improving the material removal efficiency of the gel disc. Concurrently, the SNR of *S_a_* shows improvements of 2.2% and 3.4% at 49 N and 98 N pressures, respectively, indicating that higher pressures effectively suppress surface waviness and reduce surface roughness. These findings demonstrate that a pressure of 98 N provides optimal balance between high *MRR* and superior surface quality stability.

Beyond pressure, rotational speed significantly influences polishing performance. The data show that increasing speed from 30 rpm to 60 rpm and 90 rpm enhanced *MRR* SNR by 3.6% and 11.1%, respectively, while *S_a_* SNR improved by 0.3% and 7.6%. This indicates that the increase in rotational speed leads to an increase in the number of contacts between the abrasive grains and the workpiece per unit time, and the overlap rate of the cutting trajectory rises rapidly, thereby improving the *MRR*. Higher rotational speeds help reduce the cutting residue of individual abrasive grains, making the material removal more uniform and thereby lowering the surface roughness. Therefore, in order to achieve a better polishing effect, the rotational speed is set at 90 rpm.

Comprehensive analysis of experimental data and theoretical considerations reveals that the parameter combination A2B3C3 (4 wt% TEA concentration, 98 N polishing pressure, 90rpm rotational speed) yields optimal processing performance for SiC workpieces, achieving the best compromise between *MRR* and surface quality.

(4) $${SNR}_{i}=-10log\frac{1}{r}\sum_{j=1}^{r}S_{ij}^{2}$$

(5) $${SNR}_{i}=-10log\frac{1}{u}\sum_{j=1}^{u}\frac{1}{M_{ij}^{2}}$$

### 4.4. Comparative Polishing Experiment

**Figure 11:**
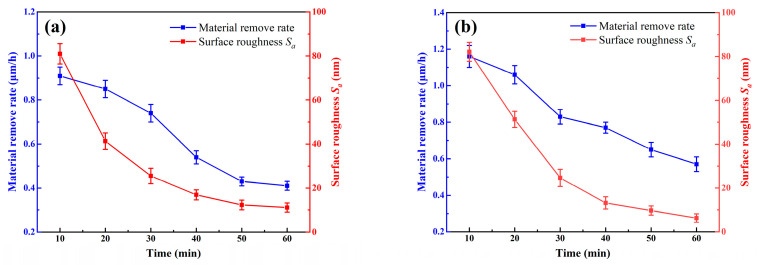
*MRR* and *S_a_* of SiC after rough polishing with different polishing solutions: (**a**) aqueous polishing solution, (**b**) TEA polishing solution.

**Figure 12:**
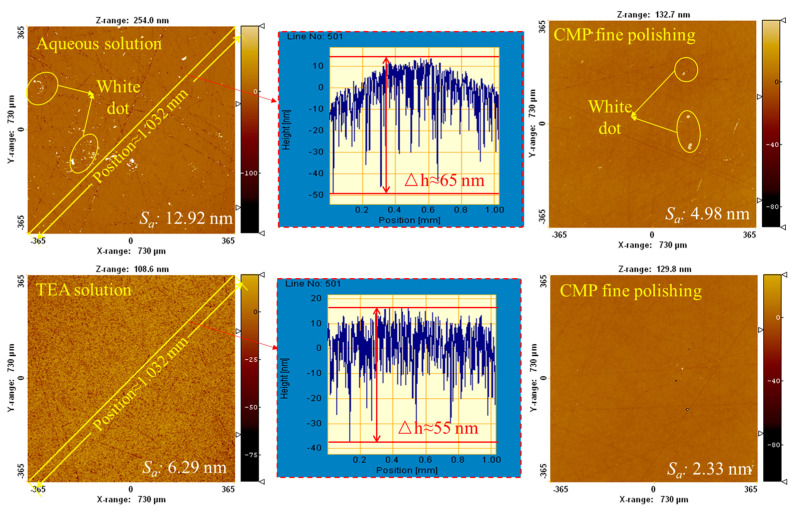
Surface morphology of SiC polished with different polishing solutions.

To systematically evaluate the impact mechanism of TEA on the self-sharpening performance of gel disc, this study designs comparative polishing experiments using pure water-based polishing fluid as the control group. Figure 11 illustrates the effects of different polishing fluid systems on surface roughness *S_a_* and *MRR* during the coarse polishing stage, while Figure 12 further elucidates the polishing mechanism through surface morphology analysis. During rough polishing, the control group using pure water-based polishing fluid exhibits significant process limitations. As shown in Figure 11a, the MRR of this group decreases sharply after 30 min of polishing. This phenomenon results from severe pore clogging on the disc surface and glazing effect caused by localized overheating: the glazed layer forms a dense coating on abrasive particles, significantly reducing the proportion of effective cutting grains and leading to substantial attenuation of mechanical removal efficiency. Surface quality analysis reveals that the glazing effect hinders effective removal of surface asperities on SiC, creating obvious white spot defect zones, with measured average *MRR* of 0.63 μm/h, surface roughness *S_a_* of 12.92 nm, and line height difference Δ*h* of 65 nm. In contrast, the TEA-containing polishing fluid system demonstrates remarkable process advantages. Figure 11b shows that although the *MRR* similarly exhibits a declining trend, the decrease is significantly more gradual, maintaining an average *MRR* of 0.85 μm/h—a 34.9% improvement over the control group. Mechanism analysis indicates that the amine groups in TEA molecules selectively react with the copper ions on disc surface through complexation, producing multiple beneficial effects: First, the complexes effectively prevent continuous formation of the glazed layer. Second, they moderately weaken the binder’s holding force on abrasive particles, promoting timely shedding of passivated grains. Third, the increased chip space significantly improves debris removal conditions. These synergistic effects maintain excellent self-sharpening performance of the gel disc, ultimately achieving superior surface quality (*S_a_* = 6.29 nm, Δ*h* = 55 nm). The surface roughness exhibited a 51.3% improvement compared to the other group.

After subsequent CMP fine polishing using polyurethane pads and silica sol slurry, the TEA experimental group finally achieves an ultra-smooth SiC surface with roughness as low as 2.33 nm (Figure 12). These results confirm the dual efficacy of TEA additive in improving gel disc self-sharpening, enhancing both processing efficiency and surface quality, providing a new process optimization direction for precision machining of hard and brittle materials.

## 5. Conclusions

This study utilizes the complexation reaction between TEA polishing solution and copper ions in the gel disc to reduce the tool’s holding force, decrease glazed areas, increase chip storage space, and ultimately achieve self-sharpening of the gel disc. The TEA-based polishing solution effectively suppresses glazing phenomena on the gel disc, enabling highly efficient workpiece polishing. The experimental conclusions obtained in this study are as follows:

(1) TEA concentration significantly influences polishing efficiency, with an optimal range (e.g., 4 wt%) balancing complexation and mechanical abrasion. This prevents glazing or excessive wear, ensuring consistent self-sharpening and machining quality. At 4 wt% TEA, the chemical-mechanical synergy is optimized, avoiding glazing or excessive corrosive wear.

(2) The best-performing parameters (TEA: 4 wt%, pressure: 98 N, speed: 90 rpm) achieve an optimal balance between surface roughness and *MRR*. Insufficient TEA concentration leads to glazing, while excessive TEA causes abrasive shedding. Moderate increases in pressure and speed enhance cutting efficiency and surface quality.

(3) TEA polishing solution effectively inhibits glazing of gel discs through complexation reactions, enhances self-sharpening performance, increases the *MRR* of rough polishing by 34.9% (0.85 μm/h), reduces the surface roughness of SiC by 51.3% (*S_a_* = 6.29 nm), and finally achieves an ultra-smooth SiC surface (*S_a_* = 2.33nm) after fine polishing. This confirms its dual efficacy in optimizing the precision processing of hard and brittle materials.

## Figures and Tables

**Table 1:** Compositional formulation of the gel disc.

Component	Granularity/μm	Solid Content/wt%
PVA + PF		15
Diamond powder	2.5	20
Electrolytic copper	3	55
Alumina powder	0.5	5
Wetting agent		≤1
Toughening agent		≤1
Others		3

**Table 2:** Experimental parameters of orthogonal experiment.

Processing Conditions	Parameters
Size of workpiece	20 × 20 mm
Size of polishing disc	Ø 100 mm
Rotational speed	30, 60, 90 rpm
Polish fluid	TEA solution
TEA concentration	1 wt%, 4 wt%, 7 wt%
Pressure	0, 49, 98 N
Flow rate	24 mL/min
Trial time	60 min

**Table 3:** Experimental parameters of comparative polishing experiment.

Processing Conditions	Parameters
Size of workpiece	Ø 100 mm
Size of polishing disc	Ø 280 mm
Polish fluid	Water/TEA solution
Rotation speed	90 rpm
Pressure	98 N
TEA concentration	4 wt%
Flow rate	24 mL/min
Trial time	60 min

## Data Availability

Data are contained within the article.
